# *'It's risky to walk in the city with syringes'*: understanding access to HIV/AIDS services for injecting drug users in the former Soviet Union countries of Ukraine and Kyrgyzstan

**DOI:** 10.1186/1744-8603-7-22

**Published:** 2011-07-13

**Authors:** Neil Spicer, Daryna Bogdan, Ruairi Brugha, Andrew Harmer, Gulgun Murzalieva, Tetiana Semigina

**Affiliations:** 1 Faculty of Public Health and Policy, London School of Hygiene and Tropical Medicine, Keppel Street, London, WC1E 7HT, UK; 2 School of Social Work, Kyiv-Mohyla Academy, 2 Skovorody Vul, Kyiv, 04070, Ukraine; 3 Department of Epidemiology and Public Health, Royal College of Surgeons in Ireland, 123 St Stephens Green, Dublin 2, Ireland; 4 Health Policy Analysis Center, Togolok Moldo 1, Bishkek, 720040, Kyrgyz Republic

## Abstract

**Background:**

Despite massive scale up of funds from global health initiatives including the Global Fund to Fight AIDS, Tuberculosis and Malaria (Global Fund) and other donors, the ambitious target agreed by G8 leaders in 2005 in Gleneagles to achieve universal access to HIV/AIDS treatment by 2010 has not been reached. Significant barriers to access remain in former Soviet Union (FSU) countries, a region now recognised as a priority area by policymakers. There have been few empirical studies of access to HIV/AIDS services in FSU countries, resulting in limited understanding and implementation of accessible HIV/AIDS interventions. This paper explores the multiple access barriers to HIV/AIDS services experienced by a key risk group-injecting drug users (IDUs).

**Methods:**

Semi-structured interviews were conducted in two FSU countries-Ukraine and Kyrgyzstan-with clients receiving Global Fund-supported services (Ukraine n = 118, Kyrgyzstan n = 84), service providers (Ukraine n = 138, Kyrgyzstan n = 58) and a purposive sample of national and subnational stakeholders (Ukraine n = 135, Kyrgyzstan n = 86). Systematic thematic analysis of these qualitative data was conducted by country teams, and a comparative synthesis of findings undertaken by the authors.

**Results:**

Stigmatisation of HIV/AIDS and drug use was an important barrier to IDUs accessing HIV/AIDS services in both countries. Other connected barriers included:

criminalisation of drug use; discriminatory practices among government service providers; limited knowledge of HIV/AIDS, services and entitlements; shortages of commodities and human resources; and organisational, economic and geographical barriers.

**Conclusions:**

Approaches to thinking about universal access frequently assume increased availability of services means increased accessibility of services. Our study demonstrates that while there is greater availability of HIV/AIDS services in Ukraine and Kyrgyzstan, this does not equate with greater accessibility because of multiple, complex, and interrelated barriers to HIV/AIDS service utilisation at the service delivery level. Factors external to, as well as within, the health sector are key to understanding the access deficit in the FSU where low or concentrated HIV/AIDS epidemics are prevalent. Funders of HIV/AIDS programmes need to consider how best to tackle key structural and systemic drivers of access including prohibitionist legislation on drugs use, limited transparency and low staff salaries within the health sector.

## Background

At the 2005 UN Summit in Gleneagles, Scotland, G8 leaders agreed "*to develop and implement a package for HIV prevention, treatment and care with the aim of [achieving] as close as possible...universal access to treatment for all those who need it by 2010*" [1]. Despite impressive efforts by global health and HIV initiatives (GHIs) such as the Global Fund to Fight AIDS, Tuberculosis and Malaria (Global Fund) to scale up funding for HIV/AIDS services, antiretroviral therapy (ART) has still not been made available to more than 5 million of the estimated 9.5 million people who need it worldwide. There are similar problems expanding HIV/AIDS prevention programmes: according to a joint WHO-UNICEF-UNAIDS report, only 30 of 92 countries providing data had introduced needle/syringe programmes and only 26 had introduced opiate substitution therapy (OST) by 2008 [2].

There is now global consensus on the need to expand access to and coverage of HIV/AIDS interventions, including prevention programmes to injecting drug users (IDUs), commercial sex workers (CSWs), prisoners and other high-risk groups [3]. The Vienna Declaration launched at the XVIII International AIDS Conference in Vienna in 2010 helped direct the world's attention towards the criminalisation of illicit injecting drug users. It highlighted the impact of criminalisation on the growing HIV/AIDS epidemics of Eastern Europe and Central Asia-regions of the world that have until recently attracted marginal interest from the global health policy research community [4-6]. Ukraine for example has the fastest growing HIV/AIDS epidemic in Europe. Kyrgyzstan and other Central Asian countries have low-level epidemics; but, without effective programmes, HIV is expected to spread rapidly [3-5,7-22] (Table 1). While the HIV/AIDS epidemic continues to grow in these countries, and many people are believed to be undiagnosed and not using essential prevention, treatment and care services, there has been insufficient empirical research on access to HIV/AIDS services outside of the generalised epidemics of sub-Saharan Africa and high income countries [23-26].

**Table 1:** Ukraine and Kyrgyzstan: selected data on HIV/AIDS epidemic and Global Fund HIV/AIDS programs

	Ukraine	Kyrgyzstan
**Epidemic type**	• Concentrated	• Low
**Number of people living with HIV/AIDS**	• 176,380 (September 2010)	• 2,718 (January 2010)
**Percentage of adult population with HIV/AIDS**	• 1.6%	• 0.13%
**Growth in HIV epidemic**	• 16.8% increase in 2006 • 5.7% increase in 2009	• 15 × increase 2001-6
**Numbers of injecting drug users**	• Estimates range from 230,000 to 360,000 (2009)	• Estimates range from 25,000 (2008) to 54,000 (2002)
**Global Fund HIV/AIDS grants**	• Round One $23,354,116	• Round Two $17,073,306
	• Round Six $131,537,035	• Round Seven $28,209,091
**Global Fund HIV/AIDS grants as proportion of total HIV/AIDS funding**	• 72.2% (2004-8)^1^	• 47% (2007)
**Clients receiving Global Fund-financed services**	• 6,070 people receiving ARVs (by Dec. 2008)^2^	• 242 people receiving ARVs (by January 2010)
	• 195,379 IDUs received preventative services (by 2009)	• 20,057 IDUs on harm reduction programs (cumulative for Round 2 grant March 2004-February 2009)
	• 33,449 female CSWs received preventative services (by 2009)	• 10,849 CSWs received preventative services (cumulative for Round 2 grant, March 2004-February 2009)

Sources: [3,9-11,15,17-22]

^1 ^Excluding out-of-pocket expenses

^2 ^By 2009, 11,900 people were receiving ARVs of which 10,787 were financed by the state budget.

Established in 2002, the Global Fund is an international financing institution, supported by a Geneva-based Secretariat, which is tasked with raising and distributing funds to support country HIV/AIDS, tuberculosis and malaria programmes. Finances are pledged by country governments, foundations and other donors and grants are made to fund control programmes in low and middle-income countries where one or more of the three diseases is endemic. Grants are awarded based on proposals prepared and submitted by multisectoral Country Coordination Mechanisms, which are meant to include the major country stakeholders: governments, civil society and development partners. A Technical Review Panel of independent international experts reviews and scores each proposal for quality and appropriateness. Where grants are approved by the Global Fund Secretariat and its governing Board, funding is awarded to and managed by one or more country Principal Recipients, which is most commonly the Ministry of Health or Finance.

By December 2010 the Global Fund had approved funding of US$21.7B for more than 600 programmes in 150 countries [27]. The Global Fund has provided substantial external resources for HIV/AIDS control to Eastern European and Central Asian countries, enabling increased population coverage of HIV/AIDS services [11-16]. In Ukraine and Kyrgyzstan, Global Fund resources represent a high proportion of total HIV/AIDS financing and is reported to have funded the delivery of services to significant numbers of clients (Table 1), although in both countries multiple donors have supported HIV/AIDS-related programmes including those focussing on IDUs. Global Fund programmes have funded both government and nongovernmental organisations to provide HIV/AIDS services in Ukraine and Kyrgyzstan. At the time of the survey, which was conducted in 2007 and 2008, government specialist AIDS Centres provided most HIV testing and treatment; government Narcology Centres provided OST for IDUs; and nongovernmental organisations (NGOs) provided preventive services including harm reduction (needle/syringe exchange), condom distribution for sex workers, awareness-raising and social support programmes for IDUs. Some of these were delivered as outreach services, and some delivered from fixed sites [11,12].

Nevertheless, despite increased funding, barriers to accessing services are substantial. In Ukraine only 32.9% of registered people living with HIV/AIDS (PLWHA) in 2007 had ever used HIV services (all types), the equivalent of 13.1% of the total estimated number of PLWHA [17]. In Kyrgyzstan, despite extensive scale up, preventive programmes had yet to reach many IDUs. It is difficult to establish the total number of IDUs; estimates suggest there were at least 25,000 IDUs in 2008, and an estimated 20,057 had ever received at least one Global Fund-financed harm reduction intervention by 2008 (cumulative) suggesting individuals were not receiving these services routinely [28]. Moreover, concerns have been expressed that in order to demonstrate rapid results in both countries, in response to funders' demand for performance-based funding, there has been a tendency to fund and implement programmes in easy-to-reach groups and to target urban areas, rather than to allocate resources equitably to more marginalized groups and to those in rural and other regions that are difficult to access [11-16], problems that are also reported more widely beyond these countries [29,30].

In this paper, we report and discuss qualitative findings from a comparative study conducted in Ukraine and Kyrgyzstan in 2007 and 2008 that aimed to shed light on the effects of scale up of funding from the Global Fund on access to HIV/AIDS services. Our focus on Global Fund supported HIV/AIDS programmes rather than programmes tackling other blood-borne viruses reflects the mobilisation of significant new global resources directed at the scale-up of HIV/AIDS programmes, and an interest among funders, policy makers and practitioners on the effects of global funding on access to these services. Our work covers HIV/AIDS prevention services provided by NGOs that target IDUs: harm reduction (needle/syringe exchange), awareness-raising, and social support programmes (outreach services and those delivered from a fixed site). We also consider HIV testing, treatment and OST provided by government service providers for IDUs in both countries.

### Conceptualizing healthcare access and utilisation

Access can be defined as the 'degree of fit' between healthcare service provision and those in need of or receiving those services. Both supply and demand side factors impact on utilisation patterns, including: *availability *(the geographical distribution of healthcare resources relative to where populations live); *affordability *(the cost of healthcare relative to clients' ability to pay); and *acceptability *(the sociocultural distance between healthcare users and providers) [31-33]. Some writers conceptualize healthcare access as being determined by multiple sets of factors or at multiple levels; for example, at individual and family levels, community and household levels, service delivery, health management, cross-sector policy, and environmental levels [34-38].

Much of the literature on access focuses on the availability and geographical distribution of health services [39-45]. Travel times and the availability of public and private transport and road networks impact on the distances populations can travel, as do populations' socioeconomic and demographic characteristics [39,44,45]. Economic and sociocultural factors also influence patterns of utilisation, as do features of healthcare delivery systems such as waiting times, opening hours, human resources, commodities and bureaucratic factors [39,44]. The economic costs of using healthcare include user fees, informally levied charges, transport costs, opportunity costs of other goods and services and the disruption of economic activities whilst seeking healthcare [44,46-48]. Sociocultural factors include communities' knowledge of health and health services, education levels, and gender relations, which can result in disparities between women's and men's healthcare access. Local attitudes and etiological beliefs about health and illness also impact on healthcare seeking [34,44,49-51]. Other writers have pointed to the importance of understanding the complexity of healthcare access that arises from factors including: the long-term engagement of services for health; the social embeddedness of factors such as stigma, or lay referrals on patterns of service use; the effects of the dynamic nature of interactions between providers and patients; and the importance of context in that an intervention that works in one setting may not work in others [52].

The majority of these studies of access have focused on healthcare generally rather than HIV/AIDS services specifically. The last decade has seen an increase in empirical research access to HIV/AIDS services, much of which has focused on the generalised epidemics of sub-Saharan Africa and high income countries [23-26]. Studies in Eastern Europe and Central Asia on HIV/AIDS and HIV/AIDS services have revealed some of the specific problems IDUs face, and how this impacts on the use of HIV/AIDS services. These include: repressive, prohibitionist drug policies linked to widespread police extortion and intimidation of IDUs and sex workers, stigma and discrimination, problems procuring and distributing harm reduction supplies that are frequently inappropriate or of poor quality, informal payments and other expenditure, compulsory registration and loss of confidentiality in service delivery settings [53-66]. However, in-depth analysis of how *multiple *barriers in combination impede access has been more limited. National level studies in Ukraine and Kyrgyzstan provide insights into patterns of use of HIV/AIDS services, although they have tended to focus more generally on the experiences of PLWHA, with some reference to IDUs [4,5,7,8,52-56].

The aim of our paper is to deepen existing knowledge on access to HIV/AIDS services. Based on fieldwork in Ukraine and Kyrgyzstan we provide an in-depth qualitative analysis of access to HIV/AIDS services by IDUs and former IDUs. Our contribution to the literature is to shed light on what are multiple, interrelated access barriers that IDUs face in attempting to use different types of government and NGO-run HIV/AIDS services including HIV prevention and treatment and drugs treatment. We identify and explore eight key sets of factors constraining access to Global Fund-financed HIV/AIDS services based on the accounts of HIV/AIDS service clients, frontline providers and stakeholders in the field of HIV/AIDS:

• stigmatisation of HIV/AIDS and drugs use;

• criminalisation of drugs use;

• discriminatory practices among service providers;

• information and client knowledge relating to HIV/AIDS and HIV/AIDS services;

• availability of commodities and human resources;

• economic barriers;

• geographical barriers;

• organisational barriers and bureaucratic constrains.

We also reflect on how different sets of factors mediate access to services provided by NGOs (needle/syringe exchange, awareness-raising and social support programmes), and how these differ from government-run services (HIV testing, treatment and OST).

## Methods

The paper draws on data from structured and semi-structured interviews conducted in Ukraine and Kyrgyzstan in 2007 and 2008 with frontline service providers, IDUs and former IDUs receiving Global Fund-supported services. The structured interviews incorporated a number of open-ended questions which we draw on in this analysis. Semi-structured interviews were also conducted with purposively sampled national and sub-national stakeholders consisting of key informants in the HIV/AIDS-related field in 2007 and 2008: government and NGO HIV/AIDS service managers, national and regional government decision makers, international development partners and Global Fund country programme implementers. The overall numbers of structured or semi-structured interviews conducted was as follows: clients receiving Global Fund-supported services (Ukraine n = 118, Kyrgyzstan n = 84); service providers (Ukraine n = 138, Kyrgyzstan n = 58); and national and subnational stakeholders (Ukraine n = 135, Kyrgyzstan n = 86). The samples are detailed in Table 2. Clients and service providers were recruited from HIV/AIDS services supported by Global Fund HIV/AIDS grants delivered by 32 government providers (HIV testing and treatment and OST) and 64 NGOs (needle/syringe exchange, awareness-raising and social support programmes) operating in three contrasting settings selected in each country for fieldwork. In Ukraine these were the capital Kyiv, Odessa (a high HIV prevalence city) and L'viv (a low HIV prevalence city). In Kyrgyzstan the study settings were the capital Bishkek, Osh and Jalalabad (high HIV prevalence cities) and Karakol (low HIV prevalence city). We interviewed at least one client and one service provider from each service sampled.

**Table 2:** Study sample sizes

	Ukraine	Kyrgyzstan	Total
**Clients**	**118**	**84**	**202**
Female	41	40	81
Male	77	44	121
Using NGO services	79	56	135
Using government services	42	28	70

**Service (frontline) providers**	**138**	**58**	**196**
NGO service providers*	88	23	111
Government service providers**	50	35	85

Stakeholders***	**135**	**86**	**221**

**Total service providers sampled**	**71**	**25**	**96**
NGO service providers*	49	15	64
Government service providers**	22	10	32

*Needle/syringe exchange, awareness-raising and social support programmes some of which were delivered as outreach services, some delivered from a fixed site

**HIV testing and treatment and OST

***Government and NGO HIV/AIDS service managers, government decision makers, international development partners and Global Fund country programme implementers.

Interviews were conducted by national researchers in Ukrainian or Russian language in Ukraine, and in Kyrgyz or Russian in Kyrgyzstan, using survey instruments designed by the authors. These were piloted, and minor adaptations were made to reflect country contexts. All fieldwork was conducted by professional national researchers trained in undertaking qualitative data collection on potentially sensitive topics including HIV/AIDS and illicit drug use. They were employed by research organisations that were independent of the HIV/AIDS services they engaged with. HIV/AIDS services included in the study-all of which were supported by Global Fund HIV/AIDS grants-were sampled purposively to enable NGO and government providers to be compared in each location. Clients of these selected services were randomly sampled. The eligibility criteria were: a) clients were currently using that particular service and b) that they had used the service for at least one month prior to the interview.

Client interviewees were recruited with the agreement of HIV/AIDS service providers who introduced potential interviewees to the researchers. The researchers described the study to the clients and elicited informed consent before proceeding to the interview. All interviews were conducted in private spaces to maintain anonymity and confidentiality which typically comprised of offices or consultation rooms within service provider premises. Staff or other clients were absent from client interviews. Individuals who might have been in need of but were not using HIV/AIDS services were not interviewed due to considerable difficulties engaging with those groups. Obstacles included locating and identifying IDUs who were circumspect about being approached by researchers unknown to them outside of HIV/AIDS service settings, since they believed this might jeopardise their anonymity thereby making them vulnerable to police arrest.

A number of data collection tools were used. The 2007 phase of the study employed client and service provider questionnaires comprising both structured questions (the results of which are reported elsewhere [11-16]) and open-ended qualitative questions the results of which are presented here. Responses to the qualitative questions were written verbatim in field notes. Stakeholder interviews took the form of in-depth qualitative interviews which were recorded and transcribed, and translated by a professional translator. The 2008 stage of the study consisted of in-depth qualitative interviews with clients and stakeholders, which were recorded and transcribed, and translated by a professional translator. Service provider questionnaires consisted of both structured and open-ended qualitative questions; responses to the latter were recorded in field notes verbatim.

Clients were asked to comment on the specific HIV/AIDS service they were using at the time of the interview; how and why they started to use the service; key access barriers and the effects of these problems on their ability to use the service effectively when they needed it. They were also invited to comment on the positive and negative features of the services; and ways the services could be improved. Service providers were asked to comment on the services they were delivering. Stakeholders were asked to comment on government and NGO-run services funded by the Global Fund and to reflect on the differences between services where possible. Both providers and stakeholders were asked to focus on their perceptions of the major barriers to access of Global Fund-supported HIV/AIDS services. While interviews did not reach saturation for all issues that emerged saturation was reached around the most important and commonly reported problems of HIV/AIDS service access, on which this paper is based.

Qualitative data from client, service provider and stakeholder interviews provided rich, explanatory insights into the problems of accessing HIV/AIDS services. The aim was to develop a better understanding of the nature and complexity of factors that obstruct access rather than to measure the scale or extent of each problem. Hence, transcripts and field notes were analysed thematically and findings elicited to produce a comparative synthesis across the two countries [67]. An investigator triangulation approach was adopted: multiple researchers contributed to analysing the findings to reduce bias and enhance the internal validity of the synthesis. The synthesis involved a five-stage process: 1) Country data in the form of transcripts and field notes were coded and cross-checked by at least two investigators from each country team; 2) cross-country findings were systematically analysed by the lead analyst and major common themes identified; 3) summaries of the major cross-country themes were presented to country teams to confirm the interpretation; 4) the lead analyst deferred to the country teams in a small number of cases where the former's interpretation differed from that of the latter; 5) the paper was drafted by the lead analyst and reviewed by country teams to confirm the study findings were accurately and coherently presented.

Ethical approval for the study complying with the Helsinki Declaration was granted by the London School of Hygiene and Tropical Medicine (reference 5078) and by relevant ethics committees in the countries where the studies took place, where such committees existed.

## Results

### Stigmatisation of HIV and drug use

Injecting drug users using HIV/AIDS services frequently reported that stigmatisation of people living with HIV and people engaged in drug use was an important barrier to using government HIV testing, treatment and OST services, and NGO preventative services in both Ukraine and Kyrgyzstan. Clients commonly reported that they were afraid to reveal their HIV-positive status, fearing a backlash from families/communities. Several clients in both countries described how they travelled substantial distances to use general clinics rather than nearby specialist government HIV/AIDS services, so as to protect their anonymity. They commented graphically on the ways stigmatisation by members of their communities and also their families, or fear of being stigmatised, had inhibited them from approaching HIV/AIDS services in the past. For example Ukrainian clients using a range of different NGO and government-run services experienced: '... *fear of HIV status being made known and violation of confidentiality*...', '... *hostile attitude of the community*...' and '... *shame*...' which reproduced a feeling of hopelessness: '... *unwillingness to address drug use or change anything in my life'*. A Ukrainian client using an NGO prevention service explained:

*Once they find out that you are HIV-positive, they chase you away; they can even fire you from a job...If you are HIV infected, they consider you to be a leper, but the disease is not transmitted through social interaction, only through blood and sexually. But people are frightened. If you say that you have HIV, none will even talk to you. They will shun you and point fingers at you...I did not tell my family that I am sick*.

The stigmatisation of drug use constituted a significant barrier to accessing NGO and government-run drugs services. For example, Kyrgyz clients indicated that many IDUs did not take up services from outreach workers in case these would reveal their drug dependence. A Kyrgyz client explained: '*If an outreach worker visits homes, a drug user hides his dependence from relatives and neighbours, he just refuses services of outreach workers'*. Stigmatisation was often sufficient to deter clients from being seen in the vicinity of narcology centres because it would be assumed by an observer that such a person was a drug user. The views of government and nongovernmental stakeholders and service providers accorded with those of clients. For example, a Kyrgyz government service provider working at a Narcology Centre explained:

...*if a person comes to a Narcology dispensary, they register him/her and this will stigmatize them for their whole life. The city is small and this information is of course confidential. However, if a person was just seen in the territory of the Narcology dispensary, people conclude that he/she has a problem; he/she is addicted or has some deviancy*.

A Kyrgyz NGO drugs service manager suggested that while IDUs were encouraged to take HIV tests many were reluctant, fearing they would be identified as HIV positive, and that parents often prevented their children who they knew to be injecting drugs from seeking HIV testing: '...*families want to hide their problems from society*...'. The interviewee suggested that some people who had received a HIV positive test result had paid service providers to supply a negative result certificate. Kyrgyz clients, service providers and stakeholders explained that while intolerance of HIV/AIDS was widespread, younger people were increasingly open and knowledgeable about HIV/AIDS, drugs and sexual practices. Ukrainian stakeholders also pointed to regional and sociocultural variations in attitudes to HIV/AIDS and sexual practices, suggesting that Orthodox and Catholic Christianity, which was strong in L'viv and other parts of western Ukraine, acted as a substantial disincentive to people seeking HIV testing for fear of community sanctions.

High levels of stigma have also been reported elsewhere. A Centre for Support for Women study [64] noted very negative attitudes to HIV/AIDS, CSWs, IDUs and MSM in Kyrgyzstan, although younger people were more tolerant than older people. The Ministry of Health of Ukraine [65] reported high levels of intolerance towards PLWHA, including among people aged 15-24 years. Our findings were consistent with these studies and revealed the negative consequences for delivering both government and NGO-run HIV/AIDS services for IDUs in both countries.

### Criminalisation of drug use

Ukrainian and Kyrgyz clients, stakeholders at national and sub-national levels and NGO and government service providers widely agreed that the criminalisation of drug use and police practices relating to the implementation of drugs laws were substantial access barriers to HIV/AIDS services. Providers and clients in both countries indicated that criminalisation posed a particular problem for NGO-run harm reduction programmes, especially needle/syringe exchange services, since small traces of drugs in used syringes constitute illegal 'storage', although the problem was also reported as common for clients carrying used injecting equipment who approached and used government-run OST services and AIDS Centres.

In both countries clients, stakeholders and NGO and government service providers reported that police officers commonly arrested drug service clients, confiscated drugs and extracted bribes for possession. For clients of needle/syringe exchange services this constituted a major disincentive to using these services, resulting in sporadic rather than regular use and acted as a particular disincentive to returning used injecting equipment. Given the possibility of being criminalised for being in possession of used syringes, this was an understandable practice. Illustrating this widely reported problem a Kyrgyz client commented: '...*it's risky to walk in the city with syringes*...'. Although return of used equipment clearly represents best practice, many programmes concentrated on distribution rather than exchange because non-return of used equipment did not impact negatively on the performance figures required by the Global Fund, which did not use this as a performance indicator.

Service providers in both countries reported that the militia (police) also regularly apprehended outreach workers, many of whom were former drug users known to the authorities. An NGO needle/syringe exchange worker in Ukraine explained that outreach workers did not visit places according to a set pattern, to avoid militia harassment, but this made it difficult for clients to know where to access their services. Service providers, stakeholders and clients also reported that police often detained IDUs using OST services when they entered or left government premises, although the frequency had reduced. A Kyrgyz client of an NGO drugs service explained that the militia regularly examined his arms to check whether he had injected recently and if so demanded bribes. He sometimes travelled to the service by taxi, at considerable expense, to avoid being stopped. Clients of substitution therapy services were required to carry a certificate stating that their methadone had been supplied legally; however, often people did not have this documentation. Several Kyrgyz clients using a range of NGO and government services commented on these problems: '*We are sick and tired of police...they pick people, [take them] to detention centres without a hearing, they beat, accuse... murder...'; '...they "plant" heroin, accuse you of a crime. I was arrested last year...'; '...they start beating at once and force you into the car...'; '...there is an example when heroin was planted to one of the guys, and he was on methadone; finally he was imprisoned'*.

IDUs using different government and NGO-run HIV/AIDS services indicated that they had developed ways to reduce the chance of being harassed or arrested by the militia. A client using an AIDS Centre in Kyrgyzstan explained: '...*a whistler is settled in the drop-in centre, he whistles [when he sees] police men...and nobody will visit this centre'*.

Some HIV/AIDS control activities financed by the Global Fund and other donors in Ukraine and Kyrgyzstan aimed to address the problems stemming from the criminalisation of drugs use both at national and local levels. NGO advocacy programmes in both countries had fostered some changes in the implementation of drugs laws in many parts of the country: new guidelines had been introduced on how militia should deal with IDUs, and programmes were launched to inform clients about their legal rights. In an attempt to promote greater understanding and tolerance, a Kyrgyz NGO provided information for clinical staff, militia and policymakers including seminars on drugs, harm reduction and HIV/AIDS with the aim of promoting greater understanding and tolerance among service providers. Furthermore, stakeholders and service providers in both countries collected data from sex workers and disseminated their findings at police forums. The challenge, however, was persuading the Ministry of Interior which, as one Kyrgyz service provider noted, 'does not recognise the existence of the problem'.

Previous studies have suggested that stigmatisation of vulnerable groups and the criminalisation of drug use in the region exacerbated risky behaviour and increased vulnerability to police human rights abuses [4,5,54,66]. A 2006 study in Ukraine, for example, revealed wide scale extortion of bribes, planting of drugs, and in some cases torture or rape of detainees and other human rights violations [54]. While recent legislative reform in Ukraine and Kyrgyzstan sought to protect these groups, in practice our findings suggest that criminalisation of drug use and police harassment remained substantial barriers to accessing essential HIV/AIDS services in 2007 and 2008, especially harm deduction services delivered by NGOs to IDU clients.

### Discriminatory practices among service providers

The study revealed discriminatory practices among HIV/AIDS service providers-especially government services-to be an important barrier to their use. Ukrainian and Kyrgyz clients indicated that government staff were often less tolerant than those of nongovernmental staff, a finding also noted by a civil society perspective report from the Open Society Institute [5]. IDU interviewees suggested that discriminatory practices of government staff of different types of HIV/AIDS services included unsympathetic attitudes to them and other vulnerable groups, the withholding of services and the demanding of informal charges. A low level of commitment and willingness to work with vulnerable populations among staff of public healthcare providers was widely perceived by clients in Ukraine and Kyrgyzstan. Many said they were circumspect about using government HIV/AIDS services, fearing they would be identified to the authorities or treated with hostility by staff they described as rude, distant and lacking understanding.

Indeed, HIV-positive clients suggested this was indicative of experiences when using general state-run healthcare services. Some had been refused hospitalisation or, having learnt that they were HIV-positive, were discharged by health workers. Potential service users avoided approaching general medical services because they were usually required to show documents including medical cards stamped to show they were HIV-positive, and there was no guarantee of confidentiality. A Ukrainian client said: '*I am scared to go to a hospital, probably, someone would recognize me, here [at this HIV/AIDS service] nobody knows me; I come here*'. In Kyrgyzstan diversity of ethnic/language groups in some areas exacerbated the difficulties clients experienced in developing effective relationships with government staff. For example a stakeholder reported that in Jalalabad in southern Kyrgyzstan-a region that has a complex ethnic/linguistic mix of Kyrgyz, Russian, Uzbek and Kazakh speakers-government service providers were often unable to communicate with clients.

Clients commented that the acceptability of different NGO and government-run HIV/AIDS services could depend upon staff attitudes. NGOs were seen as being more accessible than government services in this respect. For example a Kyrgyz service provider suggested: '...*first impression is very important for drug users; there should be such qualities as patience, tolerance'*. Similarly Ukrainian clients said: '*Nongovernmental organisations are more tolerant...more flexible and are not bound by various norms*' and: *'Here I feel safer than anywhere else...I do not feel any negative attitudes or prejudices against me. I was never refused help here'*.

A Ukrainian NGO drugs worker explained that client numbers increased as trust was built over time and people became more aware of HIV/AIDS services that were tolerant. The interviewee knew most clients by name and emphasized the importance of talking to clients so as to learn where drugs were being sold, enabling the service to more effectively target interventions. Ukrainian and Kyrgyz clients said they valued the absence of bureaucracy in accessing different NGO services. A Ukrainian client described an '...*informal and confiding atmosphere*' and the way staff were attentive, sympathetic and non-discriminating. The maintenance of confidentiality was important since most IDUs tried to conceal their drug dependence. If users believed that an NGO or government-run HIV/AIDS would not respect their confidentiality, then they would be unlikely to return. Illustrating this point a Kyrgyz client said: '*I don't want to see this outreach worker again, and will never go there again. Why did she tell my mom that I take syringes?'*.

Global Fund-supported Ukrainian and Kyrgyz NGO services targeting IDUs commonly recruited former IDUs as staff or volunteers, including former clients who were seen as having good knowledge of current clients' perspectives, thereby enabling them to build trust and provide move effective interventions. Ukrainian and Kyrgyz clients said they valued this 'peer-to-peer' principle. For example, a former client and volunteer in Ukraine explained: '...*as a former injecting drug user and being HIV positive, with a wife and children, I don't want someone else to suffer..*.'. A Kyrgyz NGO manager said: '*...their work is based on the "peer to peer" principle. So, these people know the problem from inside and it is easier for them to work, they understand more, deeper, better and they have more trust of the clients*.

Nevertheless problems were reported: a high rate of staff turnover among NGO harm reduction outreach-workers existed, with many leaving after receiving training and experience for better paid or more secure positions. Some former IDUs had reverted to drug use through coming into regular contact with current users. NGO service providers in both countries reported that the problems of staff retention were also exacerbated by the uncertainties inherent in receiving regular tranches of Global Fund grants (discussed below).

### Information and client knowledge of HIV/AIDS and HIV/AIDS services

Our study found that Ukrainian and Kyrgyz clients' access to HIV/AIDS government and NGO-run services was affected by their limited knowledge of risk factors, what HIV/AIDS services were available, and the eligibility criteria for accessing the available services. In Kyrgyzstan in particular the fact that it was possible to be tested for HIV/AIDS anonymously and free of charge was not widely known by potential clients. Kyrgyz stakeholders indicated that the level of knowledge about HIV/AIDS among the general population, particularly in rural areas, remained low.

Despite the introduction of information/educational programmes that had been supported by Global Fund HIV/AIDS programmes and other donors in Ukraine and Kyrgyzstan, clients, service providers and stakeholders agreed that many people remained unaware of the ways in which HIV was transmitted. In both countries Global Fund and other donor grants had been used to support some mass media health promotion, leaflets and other materials produced and distributed by sub-recipients, posters displayed in public spaces, and HIV/AIDS awareness lessons in some schools. In Ukraine during the 1990s media reporting of HIV/AIDS had the effect of instilling fear in society rather than providing informative commentary [5] Interviewees' accounts suggested that little had changed. One Ukrainian client said:

*... TV spots talk about danger, rather than about prevention; hence people start reacting to HIV with fear, and the whole situation is further aggravated. These spots should be modified somehow. Yes, this disease is frightening...[but] we need more explanatory information, and this information should be shared in a different manner*.

Similar problems were noted as part of the Kyrgyz Global Fund programme. A manager of a Kyrgyz NGO commented:

*The policy of prevention using fear was not right...We cultivated stigma ourselves, inspired fear...One ought to...use all resources, starting with mass media, so that people know about ways of transmission*.

Kyrgyz clients, service providers and stakeholders were critical of Global Fund-supported HIV/AIDS information programmes. A Kyrgyz stakeholder, for example, explained that social marketing for HIV/AIDS was ineffective since messages lacked cultural sensitivity outside the capital Bishkek. Often leaflets were too long, they used overly professional language, and films and posters depicted modern lifestyles and dress codes that challenged conservative views: '...*some information videos are not acceptable for our population, they show naked bodies-too explicit*...'. Hence, materials failed to reach and effectively engage marginalized groups. Another Kyrgyz stakeholder reported that providing women with information on HIV/AIDS-related issues in rural Kyrgyz communities was particularly problematic.

Clients, service providers and stakeholders suggested that peer education and referrals were important means by which communities improved their knowledge of HIV/AIDS and government and NGO HIV/AIDS services: most Ukrainian and Kyrgyz clients said that they had learned about services they were using from their peers. Kyrgyz clients using drugs services emphasised the importance of networks of drug users in delivering messages to communities. In both countries many government and NGO providers promoted peer education and referrals as ways of extending coverage. Ukrainian clients indicated that their knowledge of HIV/AIDS had improved substantially since using different NGO harm reduction services.

### Commodities and human resources

Our study suggests that shortages of medicines, commodities (including needles/syringes) and equipment (including laboratory equipment), and low quality and inappropriate commodities, were important barriers to clients receiving both government and NGO-run HIV/AIDS services. The majority of stakeholders and government and NGO service providers suggested that, while Global Fund support had allowed services to expand significantly, shortages of commodities remained a critical barrier to delivery, with reports of NGOs in Ukraine having to borrow equipment to maintain coverage. In Kyrgyzstan, clients and some stakeholders criticised the inappropriateness of some supplies procured as part of the Global Fund programme, such as the size and bore of needles and syringes supplied to service providers, which did not correspond to clients' needs (for example 2 ml syringes were preferred, whereas 10 ml syringes were generally supplied). This reduced client demand for these commodities.

Discriminatory practices and limited transparency among services impacted on access to commodities among clients. In addition to the loss of Global Fund-financed needles and syringes intended for free distribution through sale in markets, Ukrainian and Kyrgyz stakeholders also acknowledged that some government and nongovernmental organisations employed corrupt working practices, such as inaccurate record-keeping, to conceal poor levels of performance and misuse of commodities and other resources. They described an institutionalized lack of transparency among some government and NGO service providers in both countries, and underdeveloped monitoring and evaluation systems. Indeed, the monitoring and evaluation system employed by the Kyrgyz Global Fund Principal Implementing Unit (PIU) had limited means to verify activity levels reported by sub-recipients. There were infrequent or absent spot checks by PIU staff to check records, and limited *ad hoc *observations and client interviews. Stakeholders suggested that corruption was less widespread among Ukrainian HIV/AIDS services, although the practice of government health staff selling drugs such as painkillers and other supplies to drug dealers leading to shortages was still practiced.

A high proportion of Ukrainian clients perceived staff shortages as an important barrier to receiving both government and NGO HIV/AIDS services, and stakeholders in both countries indicated that low government salaries resulted in low levels of motivation, and exacerbated problems of staff retention, including international and rural-urban labour migration. Previous studies have also reported acute health worker shortages in Central Asia due to international labour migration [4]. In both countries, the Global Fund HIV/AIDS grant funded only NGOs to recruit new staff, since appointing new government staff would be considered a recurrent cost. Ukrainian stakeholders reported that some government staff had established NGOs to apply for Global Fund and other donor grants, enabling individuals to supplement their salaries.

Quantitative data collected as part of this study showed that while staff numbers among NGO HIV/AIDS services had risen, they had remained static among government services [13-16]. Stakeholders and government service providers pointed to limited financial incentives for government HIV/AIDS staff, whereas international organisations and NGOs typically paid higher salaries. In Ukraine some government health workers received supplements (including health insurance) from local government budgets. Kyrgyz government AIDS Centre staff received modest government funded salary supplements; other workers, including laboratory technicians working with blood samples, did not receive supplements.

Kyrgyz NGO service providers reported that Global Fund funding interruptions were frequently experienced by their organisations, that the problem was getting increasingly common, and that this had disrupted service delivery. In many cases this was caused by difficulties submitting quarterly monitoring reports by NGO sub-recipients on time. Most NGOs delivering needle/syringe exchange services did not stop work when financing breaks occurred, and relied on unpaid volunteers to provide services. A number of NGOs continued to distribute syringes using their own channels, violating rules in doing so. However, long interruptions in 2007-2008 forced several organisations to suspend activities, and breaks in payment of salaries forced many NGO staff to seek employment elsewhere. One interviewee explained: '*They leave for another place of work or go to Russia. When a break is too long, they just don't come back. But, to recruit new people is the same as starting again'*. These problems meant clients did not receive these services or were forced to rely on services funded by alternative donors to receive needles/syringes.

### Economic barriers

The economic transition in FSU countries in the last ten years has been traumatic. Studies have reported increased poverty and unemployment, weakened social welfare, increased domestic violence, alcoholism, intravenous drug use and sex work. These factors fuelled the HIV/AIDS epidemic and created severe financial shortfalls in the healthcare system, reducing coverage and increased out-of-pocket payments [4,7,8]. Faced with socio-economic challenges of such magnitude, Global Fund and other donor-financed HIV/AIDS services have, unsurprisingly, struggled.

Whilst notionally free to users, Ukrainian and Kyrgyz clients interviewed suggested that they frequently made additional and/or informal payments to receive commodities from government HIV/AIDS services including medicines and surgical gloves which they found expensive. The costs of obtaining necessary official documents required by government services also constituted a substantial economic barrier to using these services. Such problems were not reported by Ukrainian and Kyrgyz clients as a significant problem in utilising NGO-run services. However, observations of transactions in the markets, which were conducted as part of the Kyrgyz study, revealed that Global Fund-financed needles/syringes intended for free distribution by NGO HIV/AIDS services and some government providers were very widely available for purchase. Many clients reported that service providers, both NGO and government employees, appeared to exercise considerable discretion over whether or not to give them resources-including needles/syringes. Clients were often uncertain whether or not staff sold commodities for personal profit, or if staff were attempting to extract informal payments for commodities.

### Geographical barriers

The study revealed that there were substantial variations in geographical accessibility to HIV/AIDS services in the two focus countries. Ukrainian and Kyrgyz clients and stakeholders agreed that the main problems of geographical accessibility stemmed from the uneven distribution of both government and NGO-run HIV/AIDS services. Notable was the limited services outside larger towns/cities, but also the uneven distribution within the larger cities where the study took place. While it was beyond the study's scope to systematically interview clients living outside larger towns/cities, qualitative data point to substantial local variations in geographical accessibility. For example, clients living outside Odessa and Osh explained that distance was a substantial barrier to using both government and NGO HIV/AIDS services, exacerbated by poor public transport. Government AIDS Centres were located on the edge of built up areas in Kyiv and Odessa, reflecting the stigmatisation of HIV/AIDS, and these were poorly served by public transport in Odessa. Stakeholders and service providers reported that within larger cities such as Kyiv, Odessa and Osh, the distribution of NGOs receiving Global Fund grants was uneven: most had a history of operating within specific neighbourhoods, building trust among a small local client base but leaving many areas badly served. Clients stated that they were sometimes disinclined to travel for free needles/syringes since buying them through local retailers was less expensive than travel costs.

To address some of these problems many Global Fund-supported Ukrainian and Kyrgyz NGOs supported by the Global Fund and other donors ran outreach health promotion and needle/syringe distribution services. Basic government HIV/AIDS services had also been extended to primary and secondary government healthcare outlets in both countries where services included blood sampling for sending to AIDS Centre laboratories for HIV testing, administering antiretroviral drugs; and in some cases distributing needles and syringes. Nevertheless, clients reported that problems remained: many said they preferred to receive care through specialist government or NGO HIV/AIDS services rather than at local government clinics as it was easier to conceal their HIV/AIDS status in the former.

### Organisational and bureaucratic barriers

Clients reported that they experienced substantial organisational and bureaucratic barriers to using government HIV/AIDS services in both focus countries. They often lacked information on procedures for using these services, which varied between different providers since there appeared to be substantial discretion among individual staff, making service use unpredictable. Ukrainian clients in particular described the procedure for accessing government HIV/AIDS and indeed other government health services as complex and bureaucratic, often resulting in unanticipated costs including travelling to several different healthcare outlets, unanticipated delays and difficulties making appointments. For example a Ukrainian client explained that she was refused care by a government HIV/AIDS service because she was registered as living in another region, and the procedure of obtaining new permanent registration was lengthy and complex.

Ukrainian clients had experienced various other problems. For example: '...*in order to become a client of substitution therapy programs you need to have an HIV-positive status' *(although this requirement has now been relaxed in Ukraine) and '...*to go through rehabilitation for drug users for free, you've got to wait for 2-3 months because there is a waiting list'*. While relatively few clients in both countries felt that problems of referral between services acted as a barrier and most Ukrainian and Kyrgyz clients said that they had been referred between NGO and government services, interviewees explained that client referrals were in practice inconsistently applied and frequently consisted of informal signposting rather than formalised referral across government and NGO providers.

## Discussion: responding to access barriers-lessons for policymakers

Previous research has shown how global HIV/AIDS initiatives, including the Global Fund, have contributed to achieving international access goals by funding dramatic scale up of HIV/AIDS prevention, treatment and care services, encouraged political commitment to a highly stigmatized disease, improved linkages between government and NGO-run HIV/AIDS services, supported advocacy, and provided training [68]. Attempts to assess the extent to which universal access has been achieved have focused on the existence and coverage of HIV/AIDS services [2,3]. Nevertheless, as our study suggests, major challenges obstruct ongoing efforts to achieve the goal of universal access, especially in countries where HIV infection and risk is concentrated in marginalised and often criminalised population groups.

While HIV/AIDS service scale-up has been significant in Ukraine and Kyrgyzstan, increased service availability has not always resulted in and does not equate with increased accessibility for the populations in need of these services. Our study confirms previous work examining problems faced by IDUs in Eastern Europe and Central Asia [53-66]. Those studies and ours'-in Ukraine and Kyrgyzstan-have demonstrated the extent to which stigma and discrimination, the criminalisation of drug use resulting in heavy-handed police practices, problems supplying appropriate and high quality commodities, informal payments and other expenditure and loss of confidentiality are major barriers to IDUs accessing HIV and AIDS services. Indeed, our data suggest that accessibility problems are likely to be more extensive than many of these earlier studies show because it is evident that multiple, complex, and interrelated barriers mediate, obstruct and deter HIV/AIDS service utilisation at the level of service delivery. These include: the interrelated problems of stigma and discrimination, compounded by poor levels of information about HIV/AIDS among 'risk' groups and society as a whole; the criminalisation of drugs use, which reproduces discriminatory practices among law enforcement officers and service providers; economic and geographical barriers exaggerated by stigma, discriminatory practices and factors impacting on the regular supply of commodities; and the multiple organisational and bureaucratic barriers that clients face when seeking preventive, harm-reduction and treatment services. A key message from this study is therefore that it is *essential that debates surrounding universal access acknowledge that access is not simply determined by commodity delivery and service coverage, which represent the more easily measured performance indicators*.

In this paper we explore the specific barriers to access experienced by IDUs using HIV/AIDS services in the counties in which the study was set. These differ substantially from experiences in high-income countries where laws relating to drug use tend to be less repressive, and in generalised HIV/AIDS epidemic settings where the main mode of HIV transmission tends not to be through injecting drug use [23,24,31-51]. Indeed, we argue that it is essential to understand country contexts rather than assume access problems are universal across different settings, disease areas, client groups and service provider types. Even in other countries where injecting drug use is the main driver of the HIV/AIDS epidemic there are important differences of context which make generalisations problematic. In Thailand, for example, the evolution of the government's stance towards IDUs-from its 2003 'war on drugs' that drove IDUs underground and away from services, to its 2007 National AIDS Plan that pledged to promote and implement prevention and harm reduction services for all who needed them-has not been replicated in either Ukraine or Kyrgyzstan [69]. In Malaysia, where a similarly draconian stance towards drug use (and users) has mellowed slightly, common obstacles to access to those we identify were evident: punitive laws criminalizing behaviors; heavy-handed police responses; little attempt by government to educate communities. But religious (particularly Islamic) opposition to the concept of harm reduction proved particularly obstructive, a factor not identified as significant in our study [70].

What our study shows is that the analytical frameworks currently being employed in studies of access to general healthcare are of limited utility for understanding the complex and specific access issues facing marginalised groups such as IDUs seeking HIV/AIDS services in countries with low/concentrated epidemics. For example, earlier access studies reviewed earlier in this paper [23-26,31-52] have tended to focus on factors *within *the health sector that influenced or determined access, whereas our study shows that critical barriers to accessing HIV/AIDS services in Ukraine and Kyrgyzstan stem from *outside *the health sector. These include prohibitionist and punitive drug laws and their implementation, and the multiple stigmatisations-by officialdom, communities and even their families-of those with HIV/AIDS and of those who used illicit drugs. HIV/AIDS programmes must acknowledge and address both health service-specific *and *external political and societal factors if they are to be effective.

In countries where the HIV/AIDS epidemic is fuelled by injecting drug use, legislation and policing practices have undermined the effectiveness of Global Fund HIV/AIDS programmes. While attempts to reduce repressive police practices, some of which have been supported by the Global Fund, have made some progress, this remains a substantial problem highlighting the need for more sustained advocacy programmes at all levels, including training police officers to work more sensitively with IDUs. Ultimately, however, it is about political will. Hence, it is essential firstly to promote more effective engagement between government ministries including health, education and the interior in multi-sectoral decision making processes; and secondly to support sustained, coherent social marketing programmes aimed at reducing stigmatisation of high-risk behaviours and HIV/AIDS within society as a whole. If new funding opportunities are not used to tackle these structural and systemic drivers of the HIV epidemic, the scale of the epidemic will outstrip these countries' capacity to control it.

Another limitation of previous access studies [23-26,31-51] and studies of HIV/AIDS in the FSU region [4,5,7,8,53-66] is that they tend to focus on government healthcare while rarely acknowledging the nongovernment sector. Our study suggests that some access problems are commonly experienced in using both NGO and government-run services including stigmatisation, limited organisational transparency, low awareness of clients about available HIV/AIDS services, and economic and geographical barriers. However, other barriers to accessing government HIV/AIDS services are different to those experienced in accessing NGO services. In part this observation reflects the fact that NGOs and government providers deliver different types of services. For example needle/syringe exchange programmes-the domain of NGOs-are particularly vulnerable to police arrests and harassment. There are also critical differences in the approaches NGOs and government providers take to delivering services. The findings in our study show that NGOs are more innovative and progressive in embracing informal, non-bureaucratic approaches and non-discriminatory practices such as the recruitment of former clients as service providers. Thereby they engender tolerance, trust, the maintenance of confidentiality, and the ability to hear and understand clients' needs.

This study had a number of methodological limitations: firstly, individuals not using HIV/AIDS services were not interviewed due to considerable difficulties engaging with those groups, which constituted a sampling bias. Secondly, because sampling was limited to urban areas in the two countries, the perceptions of clients and service providers in other cities and smaller towns and rural areas were not collected. Indeed, it is a reasonable assumption that the problems of accessibility outside large cities would be greater to those experienced within them. Thirdly while our data suggest that there are some regional differences, for example particular problems experienced in Western Ukraine and Southern Kyrgyzstan and geographical variations with cities, the study did not systematically explore differences between different regions of the two focus countries. Fourthly, this study reports findings based on data collection carried out in 2007 and 2008: it therefore represents a snapshot in time in a changing policy environment in the two focus countries. Finally, while the data suggest that a similar range of access barriers are experienced in both focus countries, one should not generalise the conclusions of this study beyond Ukraine and Kyrgyzstan.

## Conclusions

If internationally agreed targets are designed to spur action, then the goal of universal access to treatment by 2010 has been moderately successful. Since 2005, scale up of additional funding from global health and HIV initiatives such as the Global Fund has made HIV/AIDS treatment and prevention available to many more people. However, as our study shows, availability of services does not equate with accessibility of services. This is a serious limitation in the policy response to HIV/AIDS services that requires immediate attention.

Our analysis shows that in at least two countries from the FSU region there are clear barriers to access facing the most vulnerable members of society. New global initiatives, such as the Global Fund, have created an opportunity for governments and NGOs to acknowledge, and respond to the rapidly growing HIV epidemic. However, HIV/AIDS remains highly stigmatised in these two countries from the FSU region, with clients reporting stigma and discrimination from families, law enforcement agencies, and notably from providers of some government health services.

Our study has contributed to the literature on HIV/AIDS in the FSU region [4,5,7,8,53-56] by providing a better understanding of what are multiple, complex barriers to accessing HIV/AIDS services in two countries in a region where limited evidence exists to date on the implementation of large-scale HIV/AIDS programmes. Conventional conceptualisations of healthcare access [23-26,31-51] need to be adapted to country contexts and, importantly, to HIV/AIDS and other disease-specific interventions if they are to be useful. Barriers to accessing general healthcare are different to those experienced accessing HIV/AIDS services, and barriers to accessing government HIV/AIDS services are different to those accessing NGO services. Indeed, there are differences between specific types of services delivered by both government and NGO providers. It is also important to recognise that in the case of HIV/AIDS, non-health system barriers are significant, including prohibitionist drugs laws and their implementation, and the multiple stigmatisations of HIV/AIDS and illicit drugs use.

While this study has started to build an understanding of the problems of accessing HIV/AIDS services in two FSU countries further research is required in order to deepen our knowledge of these problems and to help inform the development of future HIV/AIDS programmes. Large scale quantitative client surveys would be valuable in order to assess the scale of different access problems, while further qualitative research could help to better understand the health systems and structural drivers of the access problems experienced at the service delivery level. Studies are needed to explore the perspectives of people *not *using services, to compare problems of HIV/AIDS service access in urban and rural areas and between different regions of the two countries, and to compare the experience of Ukraine and Kyrgyzstan to those of other countries of the FSU region.

## Competing interests

The authors declare that they have no competing interests.

## Authors' contributions

NS led on drafting this article. NS, DB, RB, GM and TS all participated in the conception, design and execution of the study and analysis and interpretation of data. AH contributed substantially to the analysis and interpretation of data. All authors participated in manuscript writing and have read and approved the final manuscript.
